# Genomic Functional Analysis of Novel Radiation-Resistant Species of *Knollia* sp. nov. S7-12^T^ from the North Slope of Mount Everest

**DOI:** 10.3390/microorganisms12091748

**Published:** 2024-08-23

**Authors:** Xinyue Wang, Yang Liu, Zhiyuan Chen, Kexin Wang, Guangxiu Liu, Tuo Chen, Binglin Zhang

**Affiliations:** 1 Key Laboratory of Ecological Safety and Sustainable Development in Arid Lands, Northwest Institute of Eco-Environment and Resources, Chinese Academy of Sciences, Lanzhou 730000, China; wangxinyue22@mails.ucas.ac.cn (X.W.); chenzhiyuan22@mails.ucas.ac.cn (Z.C.); liugx@lzb.ac.cn (G.L.); 2 Key Laboratory of Extreme Environmental Microbial Resources and Engineering, Lanzhou 730000, China; liuyang21@nieer.ac.cn (Y.L.); 222083002012@lut.edu.cn (K.W.); chentuo@lzb.ac.cn (T.C.); 3 University of Chinese Academy of Sciences, No. 19A Yuquan Road, Beijing 100049, China; 4 Key Laboratory of Cryospheric Science and Frozen Soil Engineering, Northwest Institute of Eco-Environment and Resources, Chinese Academy of Sciences, Lanzhou 730000, China; 5 School of Petrochemical Technology, Lanzhou University of Technology, Lanzhou 730050, China

**Keywords:** Mount Everest, moraine, extreme environment microorganisms, radiation resistance, genome analysis

## Abstract

Radiation protection is an important field of study, as it relates to human health and environmental safety. Radiation-resistance mechanisms in extremophiles are a research hotspot, as this knowledge has great application value in bioremediation and development of anti-radiation drugs. Mount Everest, an extreme environment of high radiation exposure, harbors many bacterial strains resistant to radiation. However, owing to the difficulties in studying them because of the extreme terrain, many remain unexplored. In this study, a novel species (herein, S7-12^T^) was isolated from the moraine of Mount Everest, and its morphology and functional and genomic characteristics were analyzed. The strain S7-12^T^ is white in color, smooth and rounded, non-spore-forming, and non-motile and can survive at a UV intensity of 1000 J/m^2^, showing that it is twice as resistant to radiation as *Deinococcus radiodurans*. Radiation-resistance genes, including *IbpA* and those from the *rec* and *CspA* gene families, were identified. The polyphasic taxonomic approach revealed that the strain S7-12^T^ (=KCTC 59114T =GDMCC 1.3458T) is a new species of the genus *Knoellia* and is thus proposed to be named *glaciei*. The in-depth study of the genome of strain S7-12^T^ will enable us to gain further insights into its potential use in radiation resistance. Understanding how microorganisms resist radiation damage could reveal potential biomarkers and therapeutic targets, leading to the discovery of potent anti-radiation compounds, thereby improving human resistance to the threat of radiation.

## 1. Introduction

As the main peak of the Himalayas, Mount Everest, the highest mountain in the world, is subject to extreme environmental conditions, such as high radiation, low-oxygen concentrations [1], and high-temperature variations [2]. Although such extreme settings do not appear to be conducive to life, they are rich in microbial organisms that are resistant to radiation and oxidation [3] and can tolerate extreme pH and salinity levels [4]. Many microorganisms dwelling in these harsh conditions have developed survival and adaptive mechanisms to overcome these stresses [5]. For example, *Flavobacterium* sp. LB2P22^T^, isolated from the Laiku glacier on the Tibetan Plateau in China by Zhang et al., can degrade alpha-cypermethrin and has a certain degree of salt tolerance [6]. Moreover, collected from the high Arctic glacier near the settlement of Nova Oresund (Svalbard, Norway) by Xie et al., *Pengzhenrongella* M0-14^T^ was not only hydrolytically active but also grew in media containing 1–5% (*w*/*v*) NaCl [7]. Valenzuela-Ibaceta et al. isolated *Arthrobacter* EH-1B-1^T^ from Union Glacier soil in the Ellsworth Mountains, and the strain exhibited antioxidant activity and cold-acclimation response [8]. Thus, glacial ice serves as a viable ecosystem to support the survival of several microorganisms with anti-radiation and anti-oxidative activities [2]. Therefore, an in-depth study of the genomes of these strains would be helpful for developing radiation-, salt-, and low-temperature-resistant strains.

To date, only six strains have been identified in the genus *Knoellia*, including *Knoellia aerolata* DSM 18566^T^, isolated from air samples from Suwon, Republic of Korea, which could grow normally in high-salt concentrations and adapt to a wide range of pH and temperature [9]; salt-tolerant *Knoellia locipacati* DMZ1^T^ from soil in the Korean Demilitarized Zone [10]; *Knoellia remsis* ATCC BAA-1496^T^ from the air of the Regenerative Closed Life Support Module simulator system [11]; *Knoellia flava* TL1^T^ from pig feces [12]; and *Knoellia sinensis* KCTC 19936^T^ and *Knoellia subterranea* KCTC 19937^T^ from a cave in China [13]. However, none of these strains have been studied in detail in terms of their functioning, and knowledge on the genus *Knoellia* remains very sparse.

These known strains of *Knoellia* were collected from a wide range of sources: from everyday air to off-the-beaten-track caves. However, only strain S7-12^T^, which is the focus of this paper, was isolated from extreme environments, and it was more resistant to radiation than *Deinococcus radiodurans* [14], which has a high resistance to radiation and antioxidants [15]. Because strain S7-12^T^ is resistant to UV radiation, we performed an in-depth analysis of its genome and identified many genes with the drug-resistance function. Moreover, we found genes with cold tolerance and adaptation to a high-radiation environment. This is a very important discovery and proves why the strain can be isolated from extreme environmental conditions, such as those with strong radiation and oxidation in glaciers.

In May 2019, we collected moraine samples from the north slope of Mount Everest. After culturing the samples on the R2A medium, strain S7-12^T^ was obtained, purified, and cultured. To determine whether S7-12^T^ can resist radiation and oxidation, we performed experiments with various radiation and oxidation gradients. Lastly, we sequenced and analyzed its genome to understand the uniqueness of this strain and its specific functions.

## 2. Materials and Methods

### 2.1. Bacterial Isolation and Culture

On 8 May 2019, strain S7-12^T^ was collected from moraine samples on the northern slope of Mount Everest (28.02° N, 86.56° E) at 5800 m above sea level. After sampling using a sterile shovel to collect about 200 g of samples into a sterile bag, the collected samples were placed in a 4 °C incubator and transported to Everest Base Camp before the experiment samples were stored in the −20 °C refrigerator. The ecological niche is characterized by high altitude, high ultraviolet and cosmic ray radiation, low temperature, and low concentration of atmospheric oxygen [16]. Briefly, a moraine sample (5 g) was placed in a 50-mL sterile centrifuge tube with 30 mL of sterile saline (0.85%) and shaken at 180 rpm at 30 °C for 40 min. The supernatant (100 µL) was diluted to 10^−4^, dissolved in Reasoner’s 2A (R2A) agar medium [17], and incubated at 30 °C for 15 days. Strain S7-12^T^ was purified and cultured on R2A agar medium for 72 h. Reference strains *K. flava* TL1^T^, *K. sinensis* KCTC 19936^T^, and *K. subterranea* KCTC 19937^T^ were purchased from the Korean Collection for Type Cultures (KCTC) and *K*. *locipacati* NBRC 109775^T^ from the Biological Resource Center, NITE (NBRC).

### 2.2. Morphological, Physiological, and Biochemical Analysis

After 72 h of incubation on R2A agar medium, the morphological characteristics of strain S7-12^T^ were observed using an electron microscope (JSM-5600, JEOL (BEIJING) Co., Ltd., Beijing, China). The Gram reaction was determined using Solarbio’s Gram staining kit (Solarbio Cat# G1132, Beijing, China). Growth temperature tests were performed on the R2A liquid medium in the range of 10–45 °C at 5 °C intervals. NaCl tolerance tests were performed on the R2A liquid medium containing 0–10% (*w*/*v*) at 1% intervals. The growth pH range was determined using the R2A liquid medium with pH 4.0–12.0 at 1.0 pH-unit intervals. Carbohydrate utilization tests, nitrogen utilization tests, and hydrolysis tests were determined according to the methods of Shirling and Gottlieb, Williams, and Kurup and Schmitt, respectively [18,19,20]. Other enzyme activities were assayed using API ZYM strips according to the manufacturer’s instructions (biome Rieux, Lyon, France).

### 2.3. Chemotaxonomic Analysis

For the analysis of the chemical taxonomic characteristics of strain S7-12^T^ and its closely related strains *K. flava* TL1^T^, *K. sinensis* KCTC 19936^T^, *K. subterranea* KCTC 19937^T^, and *K*. *locipacati* DMZ1^T^, the strains were incubated in R2A liquid medium at 30 °C for 72 h to obtain the required cell biomass. The contents of respiratory quinones, polar lipids, and fatty acids were determined. Respiratory quinones were extracted from the dried organisms (100 mg) using a chloroform/methanol (2:1, *v*/*v*) solution and analyzed using HPLC (<37 °C) [21]. The diaminoacrylic acid isomers of the cell wall and whole-cell sugars were analyzed using Lechevalier and Lecheyalier’s [22] and Staneck and Roberts’s methods, respectively [23]. The polar lipids were extracted using a chloroform/methanol/water system via two-dimensional TLC and identified according to Minnikin et al.’s method. The samples were tested and analyzed using the Sherlock MIDI standard protocol (microbial identification system 6.2b). The peak results were determined by comparison with the database TSBA 6 (version 6.21).

### 2.4. Phylogenetic Analysis

The bacterial genomic DNA extraction kit (Omega) was used to extract the DNA from cells of strain S7-12T according to the manufacturer’s instructions. The whole genome was sequenced on the Illumina Hiseq 2000 platform with >fold coverage. The genome assembly was performed using the short sequence assembly software SOAPdenovo2 v2.04-r241 [24]. The completed genome was mapped using Unicycler version 0.4.8 [25] to assemble the third-generation sequence. During assembly, the sequence was corrected and polished with long reads using Pilon version 1.22. The assembly results of the scanning maps and chromosomal genomes were predicted using Glimmer. Based on comparisons with the genome data of strain S7-12^T^, the average nucleotide identity (ANI) was calculated based on OrthoANIu (OrthoANI), BLAST (ANIb), and MUMmer (ANIm) algorithms [26,27,28,29]. The average amino acid identity (AAI) was calculated using the online resource from the Konstantinidis group (http://enve-omics.ce.gatech.edu/aai/, accessed on 19 February 2024) [30]. The genome distances were calculated using the Genome-To-Genome Distance Calculator (http://ggdc.dsmz.de/, accessed on 19 February 2024) [31]. The dDDH results were obtained from the recommended formula 2, which was independent from the genome length and robust against the utilization of incomplete draft genomes.

The 16S rRNA gene sequencing was performed by polymerase chain reaction (PCR) using the universal primers 27F (5′-AGAGTTTGATCCTGGCTCAG-3′) and 1492R (5′-TACGGYTACCTTGTTACGACTT-3′) [32]. The sequences of both strands of the PCR-amplified 16S DNA were determined by Tsingke Company (Xi’an, China) using the dideoxy chain termination method with an ABI 3730XL Analyzer (Applied Biosystems, Waltham, MA, USA). The almost complete sequence of the 16S rRNA gene was compiled using the SeqMan software v12.3 (Lasergene) and analyzed using EzBioCloud (https://www.ezbiocloud.net/, accessed on 19 February 2024). The consensus sequences of strains belonging to the same phylogenetic group and those of other representatives of the Alphaproteobacteria were aligned using the ClustalW multiple alignment program [33]. Phylogenetic trees were constructed using the neighbor-joining [34], minimum-evolution, and maximum-likelihood [35] methods, followed by bootstrap analysis with 1000 resamplings [35] using MEGA 11 [36].

Kimura’s two-parameter model [37] was used for nucleotide substitution to estimate genetic differences. The phylogenomic tree was reconstructed based on the up-to-date bacterial core gene set according to the pipeline suggested by Na et al. [38].

### 2.5. Genomic Analysis and Prediction

The Prokaryotic Genome Annotation System (Prokka) was used to generate the protein and nucleotide sequences of the genes and annotation files (GFF3, GBK) to ensure the consistency and reliability of genome annotations and gene predictions and perform downstream genome analysis.

The gene prediction of plasmids was performed after the sequencing of strain S7-12^T^ using Glimmer version 3.02 and GeneMarkS version 4.30. The rRNAs and tRNAs contained in the genome of the strain were predicted using Barrnap version 0.4.2 and tRNAscan-SE version 1.3.1. The 16sRNAs in the genome were predicted using the 16s database and compared with the housekeeping gene database.

The NCBI prokaryotic genome annotation pipeline [39] was used to predict the tRNA genes, rRNA genes, and noncoding rRNA genes of strain S7-12^T^. The genomes were annotated using Rapid Annotation of Subsystem Technology [40]. The Kyoto Encyclopedia of Genes and Genomes (KEGG) [41], Clusters of Orthologous Groups (COG) of proteins [42], NCBI Non-Redundant Protein [43], Protein Families [44], Swiss-Prot [45], and Carbohydrate Active Enzymes databases were selected for retrieval to improve functional annotation [46]. The biosynthetic gene cluster of secondary metabolites was predicted by silicon calculation using AntiSMASH 6.0.1 (https://antismash.secondarymetabolites.org/, accessed on 19 February 2024) [47]. The statistical analyses were performed in SPSS version 16.0 [48]. The pan-genome was constructed using the Bacterial Pan Genome Analysis software [49]. The genome sequencing data of the strain S7-12^T^ was deposited in the GenBank database with the accession number.

### 2.6. Radiation-Resistance Analysis

*Escherichia coli* BL21 was used as a control strain. First, 0.5 mL of strain inoculum in the exponential growth phase (OD_600_ = 0.6) was grown in 10 mL of R2A liquid medium, placed in a 50-mL triangle bottle, and incubated at 30 °C with shaking at 200 rpm. The inoculum was diluted with saline to 10–4 once OD_600_ = 1.0 was reached. As radiation-free control, 100 μL of inoculum was diluted to 10–4 and grown on the R2A liquid medium, the other medium receiving 100 J/m^2^ UVC radiation, respectively. After 24 h of incubation at 30 °C, the number of colonies on the R2A liquid medium was counted. The radiation survival rate was calculated as follows: (*N_s_*/*N_c_*) × 100%, where *N_s_* is the number of colonies spreading irradiated inoculum on the R2A agar substrate, and *N_c_* is the number of colonies spreading radiation-free inoculum on the R2A agar substrate. All the experiments were performed in triplicate.

## 3. Results and Discussion

### 3.1. Phylogenetic Characterization Based on 16S rRNA Gene Sequencing

The full-length 16S rRNA gene sequencing and genome data of strain S7-12T were stored in the JCM/GDMCC/GenBank with accession numbers GDMCC 1.3458 and GCA_040518285.1, respectively.

By comparing the 16S rRNA gene sequences of strain S7-12^T^ in the EzTaxon database, this species was identified and classified into phylum *Actinobacteria*. The highest similarity values to strain S7-12^T^ were found with members of the genus *Knoellia*. The species closely related to strain S7-12^T^ are *K. locipacati* DMZ1^T^, *K. sinensis* KCTC 19936^T^, *K. subterranea* KCTC 19937^T^, *K. aerolata* DSM 18566^T^, *K. flava* TL1^T^, and *K. remsis* ATCC BAA-1496^T^, with 16S rRNA gene sequence similarity levels of 98.61%, 98.55%, 98.41%, 98.13%, 97.58%, and 97.16%, respectively.

**Figure 1:**
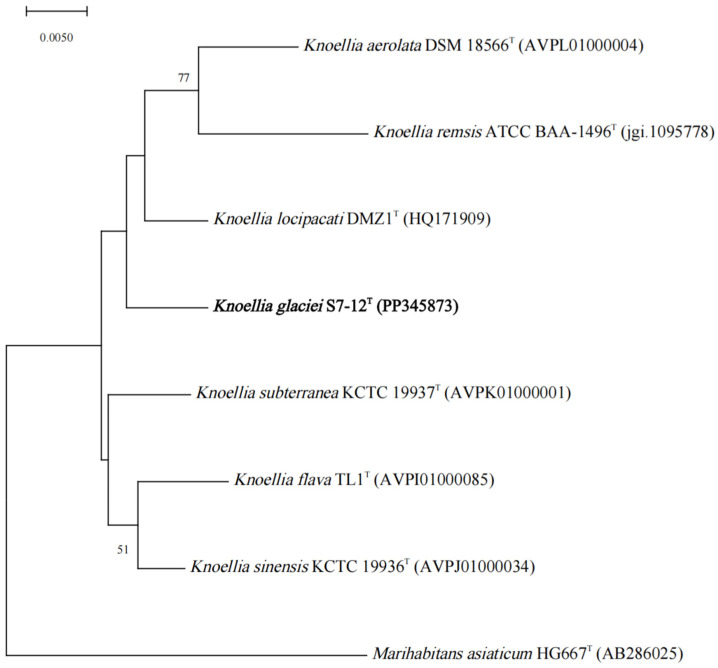
Neighbor-joining phylogenetic tree based on 16S rRNA gene sequences of the strain S7-12^T^ and the type strains of other closely related species in the genus *Knoellia* and *Marihabitans*. *Marihabitans asiaticum* HG667^T^ (AB286025) was used as an outgroup. Bar, 0.005 substitutions per nucleotide position.

**Figure 2:**
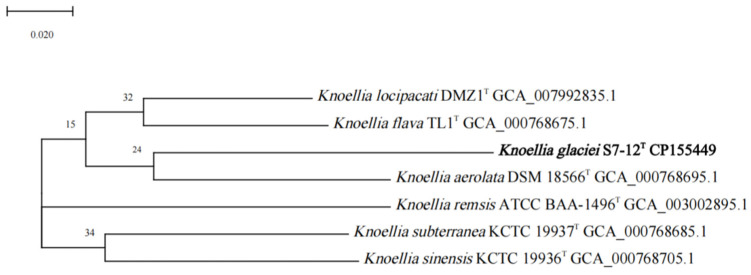
UBCG phylogenetic tree based on the up-to-date core gene set and pipeline of strain S7-12^T^ and the type strains of other closely related species in the genus *Knoellia* and *Marihabitans*. *Marihabitans asiaticum* HG667^T^ (AB286025) was used as an outgroup.

The dDDH and ANI values of S7-12^T^ with other similar strains in the genus *Knoellia* reached 22.3–24.3% and 79.31–81.60%, respectively, which were lower than the thresholds for the identification of a new species (70% for dDDH and 95% for ANI). This confirms that S7-12^T^ is a novel species [50]. The phylogenetic tree was reconstructed using four algorithms with sixteen type strains that are highly related to strain S7-12^T^ and four *Knoellia* species isolated from the northern slope of Mount Everest. A neighbor-joining dendrogram with *Marihabitans asiaticum* DSM 18935^T^ as an outgroup shows the phylogenetic position of strain S7-12^T^ (Figure 1, Figures S1 and S2). The three phylogenetic trees based on 16S rRNA gene sequences showed that S7-12^T^ forms a stable branch, suggesting that it is a member of the genus *Knoellia*. The UBCG phylogenetic tree showed that strains S7-12^T^ and *K. aerolata* DSM 18566^T^ clustered together to form a stable branch (Figure 2). This also indicates that strain S7-12T belongs to the genus Knoellia.

For the phylogenetic tree generated with UBCG using the amino acids sequences, the numbers at the nodes indicate the gene support index. Bar, 0.02 substitutions per nucleotide position.

### 3.2. Phenotypic Characterization

**Figure 3:**
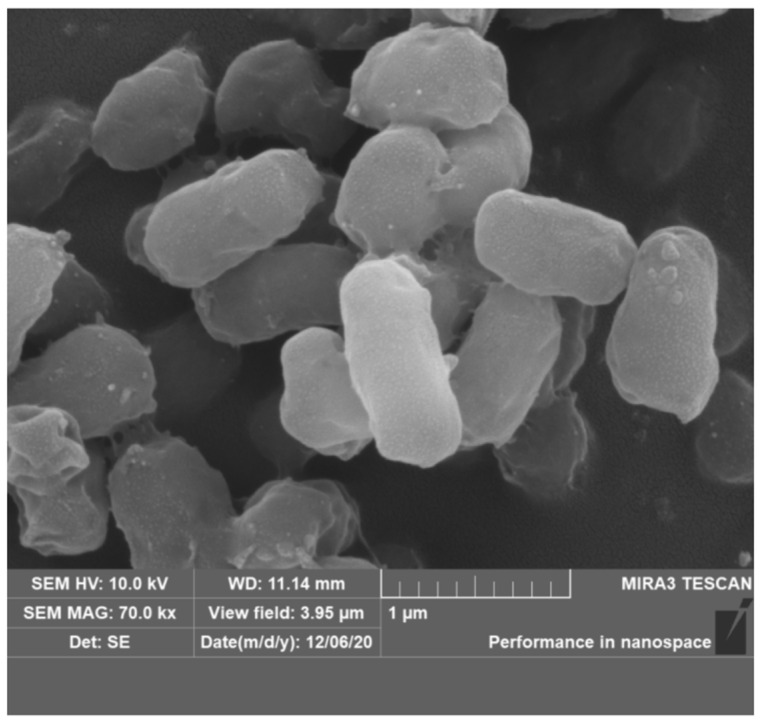
Scanning electron microscope photos of the cells of strain S7-12^T^.

After 72 h of incubation on the R2A liquid medium at 30 °C, strain S7-12^T^ formed rounded colonies with regular, raised edges and globular cells. It was colorless and opaque. The strain was Gram-negative, aerobic, non-motile, and non-budding (0.4 μm × 0.6 μm, 0.6 μm × 1.0 μm) (Figure 3).

The growth temperature range was 10–35 °C (optimum temperature at 30 °C), and the growth pH range was pH 6.0–8.0 (optimum pH at 7.0). It could tolerate 3.0% (*w*/*v*) NaCl conditions (optimum growth under 1% NaCl). Compared to *K. flava* TL1^T^, *K. aerolata* DSM 18566^T^, *K. locipacati* DMZ1^T^, *K. remsis* ATCC BAA-1496^T^, *K. sinensis* KCTC 19936^T^, and *K. subterranea* KCTC 19937^T^, strain S7-12^T^ grew in the same temperature range but a narrower pH range (Table 1).

### 3.3. Chemotaxonomic Characteristics

The major fatty acids of strain S7-12^T^ were iso-C_16:0_, iso-C_16:1_H, and C_17:1_ω8c. Of these, iso-C_16:0_ had the highest concentration in other *Knoellia* strains. By contrast, in strain S7-12^T^ iso-C_16:1_H and C_17:1_ω8c had the highest concentration (Table 2). The cell wall amino acids of strain S7-12^T^ mainly include meso-diaminopimelic acid, and the cell wall sugar components are mainly ribose (rib), glucose (glu), arabinose (ara), rhamnose (rha), xylose (xyl), mannose (man), and galactose (gal). The main polar lipids are diphosphatidylglycerol (DPG), phosphatidylglycerol (PG), phosphatidylethanolamine (PE), phosphatidylinositol (PI), phospholipids (PL1-8), an unidentified glycolipid (GL), and aminophosphoglycolipid (APGL) (Figure S3).

### 3.4. Radiation Resistance

**Figure 4:**
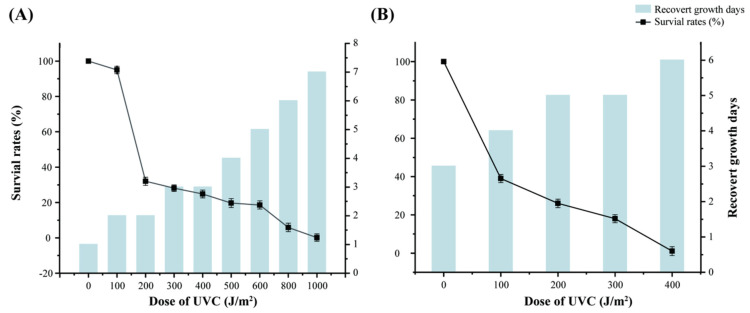
Comparison of UV irradiation resistance and days to recovery of growth between strain S7-12^T^ (**A**) and strain *D. radiodurans* (**B**).

To assess the resistance of strain S7-12^T^ to UV-NIR (UVC 254 nm) radiation, the most commonly used radiation-resistant strain, *D. radiodurans*, was chosen as reference (Figure 4). To control the variables, a 10^−4^ concentration gradient was used for both strains, and the irradiation gradient was set at 0–2000 J/m^2^. After irradiation, the survival rate of both strains decreased with an increase in irradiation dose, and strain S7-12^T^ had a higher radiation resistance than *D. radiodurans*. Notably, S7-12^T^ could survive even after irradiation with 1000 J/m^2^, whereas the control bacteria no longer survived at irradiation intensity higher than 500 J/m^2^. These results confirm the high radiation resistance of S7-12^T^.

The growth period of strain S7-12^T^ and the reference strain after irradiation was 7 days (7 d). By contrast, under normal growth conditions, the strain required only three days (3 d) to resume growth following irradiation. This indicates that a higher irradiation dose corresponds with a longer time for the strain to resume growth. The survival of the irradiated strains was lower than that of the non-irradiated strains, implying that although irradiation killed some strains, most of the strains exhibited radiation resistance.

### 3.5. Genomic Analysis

#### 3.5.1. General Genome Features

The complete genome of strain S7-12^T^ contained 4,163,720 bp, with a guanine–cytosine (GC) content of 67.81 mol%. The total number of coding sequences (CDSs) was 3955, and there were 50 RNAs, including 44 tRNAs and two sets of 5S rRNA, 16S rRNA, and 23S rRNA (Table S1). Only one plasmid was presented in strain S7-12^T^.

**Figure 5:**
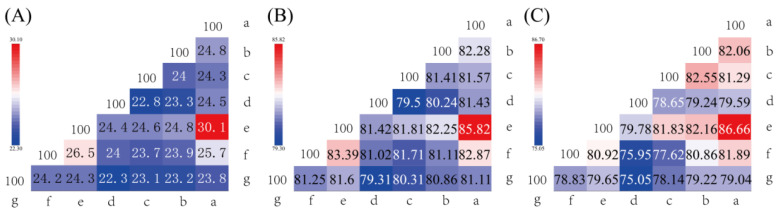
Genome comparisons of strain S7-12^T^ and its related reference strains including the dDDH value (**A**), OrthoANI value (**B**), and AAI value (**C**). Furthermore, a–g represent S7-12^T^, *K. flava* TL1^T^, *K. subterranea* KCTC 19937^T^, *K. sinensis* KCTC 19936^T^, *K. remsis* ATCC BAA-1496^T^, *K. locipacati* DMZ1^T^, *K. aerolata* DSM 18566^T^, respectively.

The AAI, ANIb, ANIm, dDDH, and OrthoANI values were calculated to identify the genomic similarities of strain S7-12^T^ to its closely related strains. The sequencing similarity values of the 16S rRNA gene were lower than 98.61% (threshold for proteobacteria) to that of the phylogenetically proximate type strain (Figure 5) [51]. The highest OrthoANI values between strain S7-12^T^ and the related strains were 81.60% (*K. locipacati* DMZ1^T^), 81.25% (*K. aerolata* DSM 18566^T^), 81.11% (*K. flava* TL1^T^), 80.86% (*K. subterranea* KCTC 19937^T^), 80.31% (*K. sinensis* KCTC 19936^T^), and 79.31% (*K. remsis* ATCC BAA-1496T), which were all lower than the 95% threshold as defined by prokaryotes [52]. The highest ANIb and ANIm values between strain S7-12T and the related strains were 81.36% and 84.97% (*K. aerolata* DSM 18566^T^), 81.74% and 85.31% (*K. locipacati* DMZ1^T^), 79.76% and 84.74% (*K. remsis* ATCC BAA-1496T), 80.59% and 84.80% (*K. sinensis* KCTC 19936^T^), 80.82% and 84.87% (*K. subterranea* KCTC 19937^T^), and 81.20% and 85.17% (*K. flava* TL1^T^), respectively, which were all lower than the 95% threshold as defined by prokaryotes [52]. The dDDH values between strain S7-12^T^ and other *Knoellia* species were 24.20% (*K. aerolata* DSM 18566^T^), 24.30% (*K. locipacati* DMZ1^T^), 22.30% (*K. remsis* ATCC BAA-1496^T^), 23.10% (*K. sinensis* KCTC 19936^T^), 23.20% (*K. subterranea* KCTC 19937^T^), and 23.80% (*K. flava* TL1^T^), which were lower than the 70% threshold as defined by prokaryotes [53]. These results indicate that strain S7-12^T^ is a novel species clustered in the genus *Knoellia*.

#### 3.5.2. COG Analysis

**Figure 6:**
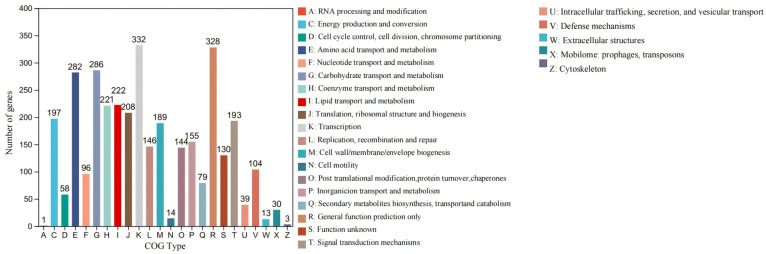
Distribution of CDS in 24 COG functional categories in strain S7-12^T^.

COGs are databases of homologous protein clusters (Table S2). COG annotation can functionally annotate unknown sequences with known proteins, identify conserved sites, and analyze their evolutionary relationships by performing multiple sequence comparisons between the sequences to be analyzed and the proteins in the COG number for comparison. A total of 3955 CDSs are distributed into 24 COG functional categories in strain S7-12^T^ (Figure 6). The major functional category includes genes that contain translation, ribosomal structure, and biogenesis (COG-J, 208 genes); transcription (COG-K, 332 genes); replication, recombination, and repair (COG-L, 146 genes); defense mechanisms (COG-V, 104 genes); signal transduction mechanisms (COG-T, 193 genes); cell wall/membrane/envelope biogenesis (COG-M, 189 genes); posttranslational modification, protein turnover, and chaperones (COG-O, 144 genes); energy production and conversion (COG-C, 197 genes); transportation of drugs/metabolites and carbohydrates (COG-G, 286 genes); amino acid transport and metabolism (COG-E, 282 genes); coenzyme transport and metabolism (COG-H, 221 genes); lipid transport and metabolism (COG-I, 222 genes); inorganic ion transport and metabolism (COG-P, 155 genes); general function prediction only (COG-R, 328 genes); and unknown function (COG-S, 130 genes). The detailed annotation results of COGs containing less than 100 genes are shown in Figure 6. Within these gene sequences, we have identified those that can form multidrug transporter proteins, resistance proteins, and hydrogen peroxide reductase.

The analysis of the COGs of S7-12 and similar strains within the genus *Knoellia* revealed significant differences in some functional abundances (Figure S4).

#### 3.5.3. Pan-Genome Analysis

The pan-genome represents the entire genetic composition of a species and is the gene pool of all strains of the species. It consists of three main components: core genes, dispensable genes, and unique genes. Pan-genomes can be further categorized into closed-type or open-type pan-genomes [54]. When the number of the sequenced genomes increases with an increase in the size of the pan-genome of a species, the genus has an open-type pan-genome; otherwise, when the number of sequenced genomes increases and the size of the pan-genome of a species increases only up to a certain extent and then converges to a certain value, the genus has a closed-type pan-genome. As a branch of comparative genomics, pan-genome analysis examines the bacterial genome from the perspective of the population and the characteristics of bacterial genome dynamics so as to evaluate the dynamic changes in bacterial genomes during evolution. The file GFF3 derived from Prokka allows for pan-genomic analysis using the Roray [55] pipeline.

We compared the core genes, dispensable genes, and unique genes from the pan-genome of this strain with the database of the essential gene (DEG) (http://www.essentialgene.org/, accessed on 19 February 2024) using the BLASTN (E − value = 1 × 10^−5^) [56]. Data accessed on 29 February 2024. The overlap between the genes and underlying genes was assessed using homogeneity scores and bits [57].

To gain a more detailed understanding of the genomic characterization and function of S7-12^T^, we performed the pan-genomic analysis after COG analysis. The Heaps’ law modeling analysis can be used to obtain an estimate of the parameter α to determine whether the pan-genome is open or closed [58]. From the equation in Figure S5 and the trend of the curve, parameter α equals 0.550, which is less than the threshold value of 1.00; thus, it is an open-type pan-genome (Figure S5).

**Figure 7:**
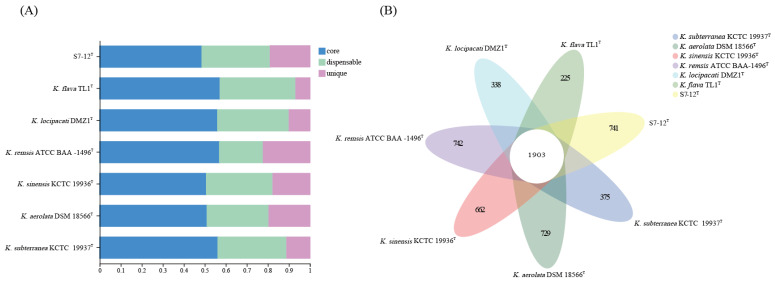
Comparisons of orthologous protein groups in S7-12^T^ and six related *Knoellia genomes*. (**A**) Percentage of core, dispensable, and unique genes in each of all eight genomes. (**B**) Venn diagram displaying the number of core and unique genes for each of the S7-12^T^ and related type strains.

Generally, gene clusters are classified as core, dispensable, or unique. A core gene cluster is a conserved gene family common to all samples of the same genus; a dispensable gene cluster refers to a gene cluster present in two or more samples at the same time; and a unique gene cluster refers to a gene cluster present only in one sample (Figure 7A). A comparative analysis based on homologous proproteomes identified 1903 core genes present in all seven *Knoellia* genomes (Figure 7B), accounting for the largest proportion. Among them, *K. subterranea* KCTC 19937^T^ has the highest core genome content, reaching up to 56% (Figure 7A). This indicates that the percentage of common functional proteins is relatively high in all types of species. The proportion of unique genomes in strain S7-12^T^ was relatively large compared to that of the other genomes in the genus *Knoellia*, which accounted for approximately 22%, the highest proportion of unique genomes among the remaining six, excluding *K. remsis* ATCC BAA-1496^T^ (Figure 7A).

Unique gene clusters play an essential role in predicting potential gene clusters that cannot be identified by traditional methods [48]. In a strain, the core genes represent the commonality in the strain, whereas the unique genes represent the distinct characteristics. Functional differences across strains can be compared based on the unique genes [59].

**Figure 8:**
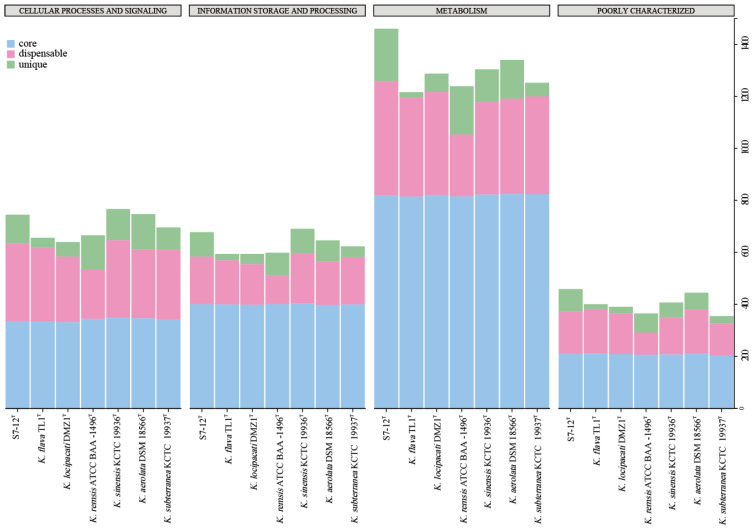
Classification of COG functions annotated to different pan-genomes in the genus *Knoellia*.

The seven species belonging to the genus *Knoellia* also have a different proportion of unique genes. The differences in the size of different genomes may affect the number of unique genomes. As can be seen in Figure 6, most of the COG functions of all strains in the genus *Knoellia* are expressed as metabolic functions. Among these pan-genomes, strain S7-12^T^ has the largest number of unique genomes compared to other similar strains in the same genus, which proves the importance of studying it. Based on the literature, the other six strains in the genus *Knoellia* originated from environmental samples of air [9], feces [12], soil [13,60], and fildes bay [61], suggesting that the genus can grow in specific environments. Furthermore, the core genome of strain S7-12^T^ is enriched in genes involved in metabolic functions, which likely contributes to its ability to utilize a wide range of nutrient sources and adapt to diverse ecological niches. This metabolic versatility could be a significant factor in the competitive advantage of strain S7-12^T^ over other strains in the genus *Knoellia* (Figure 8).

We also performed a systematic gene function analysis of all strains of the genus *Knoellia* using the KEGG database (Figure S6). A large proportion of biological processes are in the metabolism, organismal systems, human diseases, environmental information processing, cellular processes, and genetic information processing. Strain S7-12^T^ has a higher proportion of biological processes, especially involving biometabolic pathways, than the other strains in the genus *Knoellia*. This suggests that the energy drive from biometabolism is high in S7-12^T^. We speculate that this helps in DNA repair and provides higher resistance to radiation [62].

Homologous proteins are defined as proteins with similar amino acid sequences and exercise similar or identical functions. To elucidate the similarities and differences between strain S7-12^T^ and the other species in its genus, we classified the number of pan-genomes and functional genes among different *Knoellia* strains (Figure 7). A total of 1620 proteins associated with annotated genes form the core genome in all members of the genus *Knoellia*, and each member has its unique genes, except for unknown genes. In the core genome, 739 genes are responsible for functional categories and metabolism-related functions, 342 for information storage and processing machinery processing functions, 371 for cellular processes and signaling functions, and 222 are poorly characterized. Among these genes, we identified several radiation-resistant DNA repair genes, including the recombinational DNA repair protein *RecO*, a critical component of the *RecF* pathway [63]; the multifunctional *RadA*/*RecA* recombinase; and the alkylated DNA repair dioxygenase *AlkB* [64,65]. These findings suggest that strain S7-12^T^ possesses a robust DNA damage-response mechanism, which is essential for its survival in environments with elevated levels of ionizing radiation. Among the DNA repair genes we also found the ssb gene, which binds to and repairs broken single-stranded DNA in the early stages of damage repair [15]. Similarly, the genes expressing DNA repair function in strain S7-12^T^ are UvrA, UvrB, and UvrC, and the UvrABC pathway, in which these three genes are involved, provides a great help for nucleotide excision repair (NER) in the strain [66]. The gene encoding the mismatch repair enzyme MutL [67] also plays a large role in DNA repair in S7-12^T^, as do mutS, and mutH, but unfortunately, we did not find these genes in this strain.

We also identified gene sequences associated with heat-shock response proteins, such as *IbpA* [68], *HSP-20* [69], *HSP*-*70* [70], *HSP-90* [71], and the *CspA* family [72,73], and gene fragments that may express antioxidant capacity, such as *choD*, which expresses the oxidoreductase capacity of GMC [74], the DyP-type peroxidase family, DyP [75]; the cytochrome bd family, *cydA*/*cydB* [76], and cytochrome C [77]. Their presence indicates that strain S7-12^T^ has evolved a sophisticated stress-response system to adapt to a broad range of environmental stresses.

Overall, the unique genome of strain S7-12^T^ revealed a diverse set of gene sequences, including 520 genes related to cellular processes and signal transduction, 513 genes involved in information storage and processing, 1107 genes dedicated to metabolic functions, and 325 genes of unknown function. We also predicted 329 virulence genes and 240 resistance genes in the genome of S7-12^T^ (Figure S7). As seen from the two prediction maps, the genes related to nutrient/metabolic factors, immunomodulatory factors, peptide antibiotics, and macrocyclic endolipid antimicrobials account for a relatively large number of genes.

**Figure 9:**
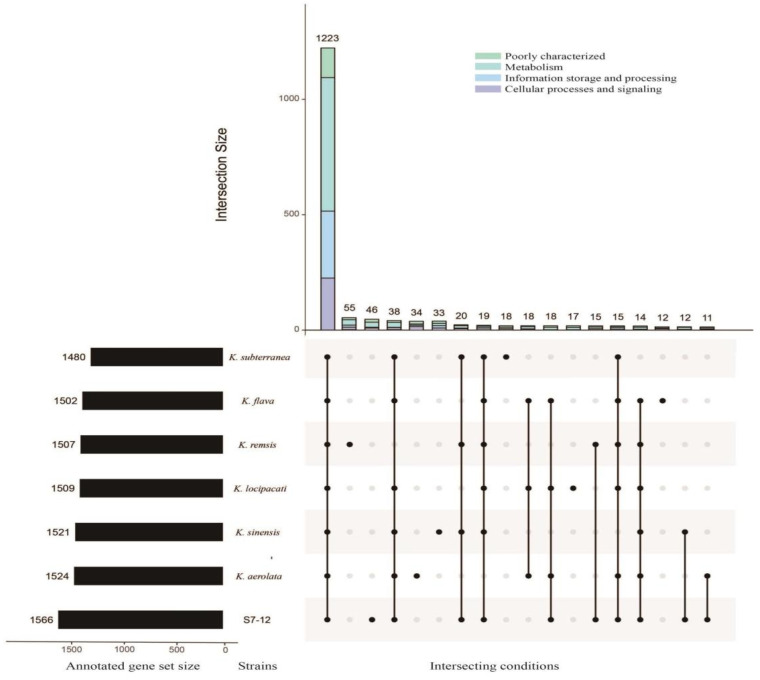
The number and functional gene classification of pan genomes between different *Knoellia* strains. The upset plot shows the number and functional classification of the core and unique genes in different *Knoellia* strains. The bar chart above represents the number of core and unique genes contained in each type of group. The strip at the bottom left represents the total number of genes in different *Knoellia* strains. The dot and line at the bottom right represent the types of different combinations (where only values above 10 and annotated genes are shown; further, unknown genes were not shown).

A further comparison of the virulence genes with those of the other bacteria within the same genus reveals a distinct family of *MntABC* genes [78] that express metal-transporter proteins, an L-methionine-binding lipoprotein (*MetQ*) [79] linked to immune evasion in gonococcal pathogenesis, and a specific *SpoVK* [80] phage motif sequence. In addition, the number of genes responsible for metabolism is the largest for all bacteria in the genus *Knoellia*, and strain S7-12^T^ has the highest number of genes with metabolism and human disease functions among all the other strains (Figure 9). These findings prove why we can find more fragments of genes concerning disease resistance and drug resistance in the genome of S7-12^T^.

#### 3.5.4. Horizontal Gene Transfer Analysis

Genomic islands (GIs) are a common type of horizontally transferred element (Table S3). They are classified according to the functions of the genes they contain, such as virulence islands, resistance islands, metabolic islands, and symbiotic islands. In addition to several core and homologous proteins that have similar functions and structures to proteins in other known strains, S7-12^T^ contains non-homologous proteins that may not have directly corresponding homologues in other known strains. The presence of non-homologous proteins may represent unique biological properties or adaptations, allowing strategic survival or competitive advantage in a particular environment.

The identification of non-homologous proteins indicates the occurrence of horizontal gene transfer events in S7-12^T^. We identified 13 GIs in S7-12^T^, containing 269 genes ranging from 73–4233 bp in length. According to the gene function analysis of GIs, most of these known functional genes are involved in cellular metabolism and membrane transport functions. Predictive gene function annotation of the GIs of S7-12^T^ revealed that S7-12^T^ has several gene sequences that can be expressed as multidrug resistance proteins *sugE* [81,82], *cysE* [83], *nisC* [84], *dinB* [85], *sprC* [86], *csoR* [87], *trkA* [88], lanthionine synthetase C-like protein *nisC* [84], proteins that can bind potential drug-binding targets *cysK* [89], and related cation transporter proteins.

In addition to these horizontally transferred genes with drug-resistance function, S7-12^T^ contains fragments of the genes *cspA*, *rpoE* [90], *sigB* [91], *resB* [92], and *recF*, indicating its potential antioxidant capacity. These genes may contribute to the survival of the bacterium in extreme environmental conditions. This gene sequence is not found in other bacteria of the same genus, suggesting that it was acquired through horizontal transfer to adapt to extreme environments.

We also found genes *cphA* and *cphB* [93] that can synthesize cyanobactin, gene family *aroK* and *aroL* [94] that can synthesize mangiferic acid, and genes *FitA* and *FitB* [95] that can produce toxin–antitoxin factors. In addition, we identified a unique gene family, namely, the BtpA/SgcQ, that can be used as reference for the treatment of drug-resistant bacterial infections [96]. Mangiferic acid is not only an intermediate metabolite in the synthesis of aromatic amino acids in *E. coli* but is also a synthetic precursor of anti-influenza drugs [97]. The antitoxin usually acts in conjunction with the toxin, which exerts toxic effects to inhibit bacterial growth, while the antitoxin can neutralize the toxicity. The interaction between the two can play a role in regulating the bacterial growth state [98]. Overall, the analysis of the predicted gene function showed that S7-12^T^ contains many drug-resistance and toxicity genes, implying its application value for drug development.

The radiation resistance of strains can be harnessed for bioremediation, significantly mitigating long-term hazards to human health and ecosystems. Amidst the escalating global challenge of antibiotic resistance, the study of bacterial resistance mechanisms in strains is pivotal for the development of novel antibiotics and therapeutic strategies, which is crucial to combat resistant infections and safeguard public health. In summary, the radiation and antibiotic resistance capacities of strains are at the forefront of biological research. By thoroughly investigating and judiciously applying these strains, breakthroughs in various fields are anticipated, contributing substantially to the advancement of human society.

## 4. Conclusions

This is the first study to describe the novel bacterial strain *Knoellia* S7-12^T^ isolated from the north slope of Mount Everest. To date, only six bacterial strains from this genus have been reported. Its mechanism of radiation resistance and genomic function were investigated under extreme environmental stresses. Multidrug-resistance, pathogenicity, and antimicrobial genes, including *cysE*, *nisC*, *sugE*, *dinB*, *sprC*, *csoR*, and *trkA*, were identified. In addition, strain S7-12^T^ contains many genes for radiation protection and cold tolerance that are not expressed in other species in the genus *Knoellia*, including *rpoE*, *sigB*, *resB*, *CspA*, and other gene families. Therefore, we speculate that this is why this strain can be isolated at high altitude in a cold-, radiation-, and oxidation-resistant environment. Owing to the expression of radiation-resistant genes, this bacterium has an increased chance of survival in a high-radiation environment. Thus, this novel strain provides opportunities for developing radiation-resistant drugs.

In addition, the gene family BtpA/SgcQ can be used as a reference for the treatment of drug-resistant bacterial infections. This gene family plays an important role in cell physiological processes and may be a potential target for the development of drugs in the future.

Overall, the experimental and genomic analysis demonstrated that the strain S7-12^T^ can resist radiation. Our findings provide the theoretical foundation for the development and application of anti-radiation drugs.

### Description of Knoellia glaciei sp. nov.

*Knoellia glaciei* sp. nov. (gla.ci. e’i. L. gen. n. *glaciei* of ice, referring to the frozen environment from which the type strain was isolated).

Cells are non-motile and non-spore-forming, growing as irregular spheres (0.5–0.7 µm in diameter) or rods (0.4–1.0 µm in diameter), appearing singly, in pairs, or clusters. Single colonies appear yellowish, round, smooth, and were raised in R2A medium for 72 h. Grow aerobically at 10–45 °C (with optimum growth at 30 °C), pH 6.0–8.0 and in 10% NaCl (with optimum growth at pH 7.0 and 1% NaCl). Positive for peroxidase but negative for oxidase. Can reduce nitrate to nitrite. Tween20, Tween80, gelatin, and starch are hydrolyzed, but urea is not. Assimilated L-arabinose, D-fructose, D-galactose, D-glucose, D-mannitol, D-raffinose, and sucrose and can weakly utilize D-xylose. Alkaline phosphatase, esterase (C4), N-acetyl-β-alkaline phosphatase, esterase (C4), N-acetyl-β-glucosaminase, and esterase lipase (C8) are detected but not lipase (C14) and acid phosphatase. The major cellular fatty acids are iso-C_16:0_H, isoC_16:0_, and C_17:1_ω8c. Characteristic cell wall sugar components are ribose and glucose. Polar lipids include diphosphatidylglycerol (DPG), phosphatidylglycerol (PG), phosphatidylethanolamine (PE), phosphatidylinositol (PI), eight phospholipids (PL), aminophosphoglycolipid (APGL), and an unidentified glycolipid (GL).

The *K. glaciei* type strain S7-12^T^ (=KCTC 59114^T^ =GDMCC 1.3458^T^) was isolated from the moraine of the north slope area of Mount Everest (28.02° N, 86.56° E), PR China. The G+C content of the genomic DNA of strain S7-12^T^ was 67.8 mol%. The full-length 16S rRNA gene sequence and genome data of strain S7-12^T^ were stored in JCM/GDMCC/GenBank with accession numbers KCTC 59114, GDMCC 1.3458, and GCA_040518285.1, respectively.

## Figures and Tables

**Table 1:** Phenotypic characteristics of strain S7-12^T^ and their closely related type strains in the genus *Knoellia.* Strains: 1. S7-12^T^; 2. *K. flava* TL1^T^; 3. *K. aerolata* DSM 18566^T^; 4. *K. locipacati* DMZ1^T^; 5. *K. remsis* ATCC BAA-1496^T^; 6. *K. sinensis* KCTC 19936^T^; 7. *K. subterranea* KCTC 19937^T^. ND, not detected. +, reacts positively. −, reacts negatively.

Characteristics	1	2	3	4	5	6	7
Isolation source	Moraine	Soil	Air	Soil	Pig manure	Air	Soil
Colony Color	White	White	White	White	White	White	White
Growth temperature (°C) range (optium)	10–45 (30)	28–37 (28)	10–35 (30)	10–35 (30)	4–42 (28)	15–45 (25)	28–37 (28)
pH range (optium)	6–8 (7)	(5–9)	5–9 (6–7)	6–9 (7–8)	4–10	6–8 (7)	(5–9)
NaCl tolerance range (optium) (%, *w*/*v*)	0–10 (1)	(4)	0–2 (1)	0–5 (1)	0–5(0)	ND	(4)
Oxidase activity	−	−	−	−	−	−	−
Production of H_2_S	+	+	ND	ND	+	−	+
Reduction of nitrate	+	+	+	ND	+	−	+
Hydrolysis of:							
Urea	−	w	−	ND	−	−	W
Tween 20	+	ND	ND	ND	+	ND	ND
Tween 80	+	+	+	ND	+	+	+
Gelatin	+	+	+	ND	+	+	+
Starch	+	+	+	+	+	ND	+
Utilization as carbon sources							
L-Arabinose	+	ND	−	ND	−	−	ND
D-Fructose	+	+	+	+	ND	+	−
D-Glucose	+	ND	+	+	+	−	ND
D-Lactose	ND	ND	ND	ND	ND	−	ND
D-Galactose	+	+	w	+	ND	+	W
D-Mannitol	+	+	+	+	+	ND	−
Sucrose	+	w	+	+	+	+	−
D-xylose	W	−	w	w	ND	+	−
Enzymatic activity							
Alkaline phosphatase	+	+	+	+	+	ND	+
Esterase (C4)	+	+	+	+	+	ND	+
Lip esterase (C8)	+	+	+	+	+	ND	+
Lipase (C14)	−	w	−	+	w	ND	W
Acid phosphatase	−	w	−	+	+	ND	W
Naphthol-AS-BI-phosphate hydrolase	+	+	ND	+	+	ND	+
N-acetyl-β-glucosaminase	−	−	−	−	−	−	−
α-Mannosidase	ND	−	−	ND	ND	ND	−
Motility	−	−	−	−	−	ND	−
Spore formation	−	−	−	−	−	−	−
Major fatty acids	iso-C_16:0_H, isoC_16:0_ and C_17:1_ω8c	i-C_15:0_, i-C_17:0_, i-C_16:0_ and ai-C_17:0_	iso-C_16:0_, C_17:1_ω8c and iso-C_15:0_	iso-C_16:0_, iso-C_15:0_ and iso-C_14:0_	iso-C_16:0_, iso-C_15:0_ and C_17:1_ω8c	iso-C_16:0_, C_18:0_ and C_18:1_	i-C_15:0_, i-C_17:0_, i-C_16:0_ and ai-C_17:0_
DNA G+C content (mol%)	67.8	68.0–69.0	73.0	72.6	70.9	69.2	68.0–69.0

**Table 2:** Whole cellular fatty acids composition of S7-12^T^ and the closely related type strains of the genus *Knoellia*. Strains: 1. S7-12^T^; 2. *K. flava* TL1^T^; 3. *K. aerolata* DSM 18566^T^; 4. *K. locipacati* DMZ1^T^; 5. *K. remsis* ATCC BAA-1496^T^; 6. *K. sinensis* KCTC 19936^T^; 7. *K. subterranea* KCTC 19937^T^. ND, not detected. All data were obtained in this study.

Fatty Acids (%)	1	2	3	4	5	6	7
iso-C_13:0_	ND	ND	0.6	ND	ND	ND	ND
C_14:0_	0.1	ND	0.6	ND	1.0	0.3	0.3
iso-C_14:0_	3.2	8.3	2.5	10.6	0.8	7.3	5.1
C_15:0_	ND	3.1	ND	3.9	ND	ND	1.7
iso-C_15:0_	3.3	15.9	15.5	12.6	9.0	11.0	6.4
anteiso-C_15:0_	0.3	ND	1.8	ND	ND	0.7	1.8
C_16:0_	0.9	1.2	1.8	1.4	5.8	1.3	2.1
iso-C_16:0_	26.3	33.6	20.7	32.6	13.7	46.6	37.2
iso-C_16:1_H	17.1	ND	0.5	1.1	ND	1.0	1.6
C_17:0_	0.4	5.5	9.5	6.1	6.7	0.6	1.1
C_17:0_ 10-methyl	ND	3.7	1.0	9.8	ND	4.8	13.0
C_17:1_ω8c	26.8	11.5	18.5	9.4	ND	0.6	6.6
iso-C_17:0_	0.2	2.1	4.4	ND	6.1	8.1	2.9
iso-C_17:1_ω9c	0.5	1.7	2.5	ND	ND	11.4	6.2
anteiso-C_17:0_	0.5	ND	6.7	ND	1.8	1.7	3.2
anteiso-C_17:1_ω9c	0.5	ND	1.9	ND	ND	ND	ND
C_18:0_	0.6	ND	1.1	ND	12.9	ND	2.1
C_18:1_ω5c	ND	ND	ND	ND	ND	ND	0.8
C_18:1_ω7c	ND	ND	ND	ND	ND	ND	1.2
C_18:1_ω9c	9.6	2.1	5.2	ND	ND	ND	1.5
iso-C_18:0_	0.3	1.2	1.1	ND	2.4	2.1	2.0
C_19:0_	ND	ND	ND	ND	ND	ND	ND
TBSA	ND	ND	ND	ND	ND	ND	0.7
C_14:0_2-OH	ND	ND	ND	ND	ND	ND	ND
C_15:0_2-OH	ND	ND	ND	ND	ND	ND	ND
C_16:0_2-OH	ND	ND	ND	ND	ND	ND	1.3
C_17:0_3-OH	ND	ND	ND	4.1	ND	ND	ND
iso-C_17:0_3-OH	ND	ND	3.4	ND	ND	1.9	ND
Summed Feature 3	5.2	1.6	1.6	ND	ND	1.3	2.1
Summed Feature 6	2.1	2.5	2.3	ND	ND	ND	ND

## Data Availability

The raw data supporting the conclusions of this article will be made available by the authors on request.

## Supplementary Materials

The following supporting information can be downloaded at: https://www.mdpi.com/article/10.3390/microorganisms12091748/s1, Figure S1: Minimum-evolution phylogenetic tree based on 16S rRNA gene sequences of the strain S7-12^T^, and the type strains of other closely related species in the genus *Knoellia* and *Marihabitans*; Figure S2: Maximum-likelihood phylogenetic tree based on 16S rRNA gene sequences of the strain S7-12^T^, and the type strains of other closely related species in the genus *Knoellia* and *Marihabitans*; Figure S3: Polar lipids profile of strain S7-12^T^; Figure S4: Comparison of COGs functional abundance between strain S7-12^T^ and similar strains in its genus; Figure S5: Characteristic curves of the pan-genome and core genome of S7-12^T^; Figure S6: Phylogenetic functional analysis of all strains of the genus *Knoellia* using the KEGG database; Figure S7: Virulence genes (A) and resistance gene prediction (B) of strain S7-12^T^; Table S1: General genomic characteristics comparison of strain S7-12^T^, and its closely related species; Table S2: The description of COG type; Table S3: Features of the GIs found in the genome of S7-12^T^.
