# Supplementary material for: Genomic Functional Analysis of Novel Radiation-Resistant Species of Knollia sp. nov. S7-12T from the North Slope of Mount Everest
Source: Microorganisms. 2024 Aug 23;12(9):1748. doi: 10.3390/microorganisms12091748 (PMC11433714; doi:10.3390/microorganisms12091748)
Supplement: Supplementary file 1 [file microorganisms-12-01748-s001.zip › microorganisms-3168135-supplementary.pdf]

Figures

Figures S1-S7

Tables S1-S3

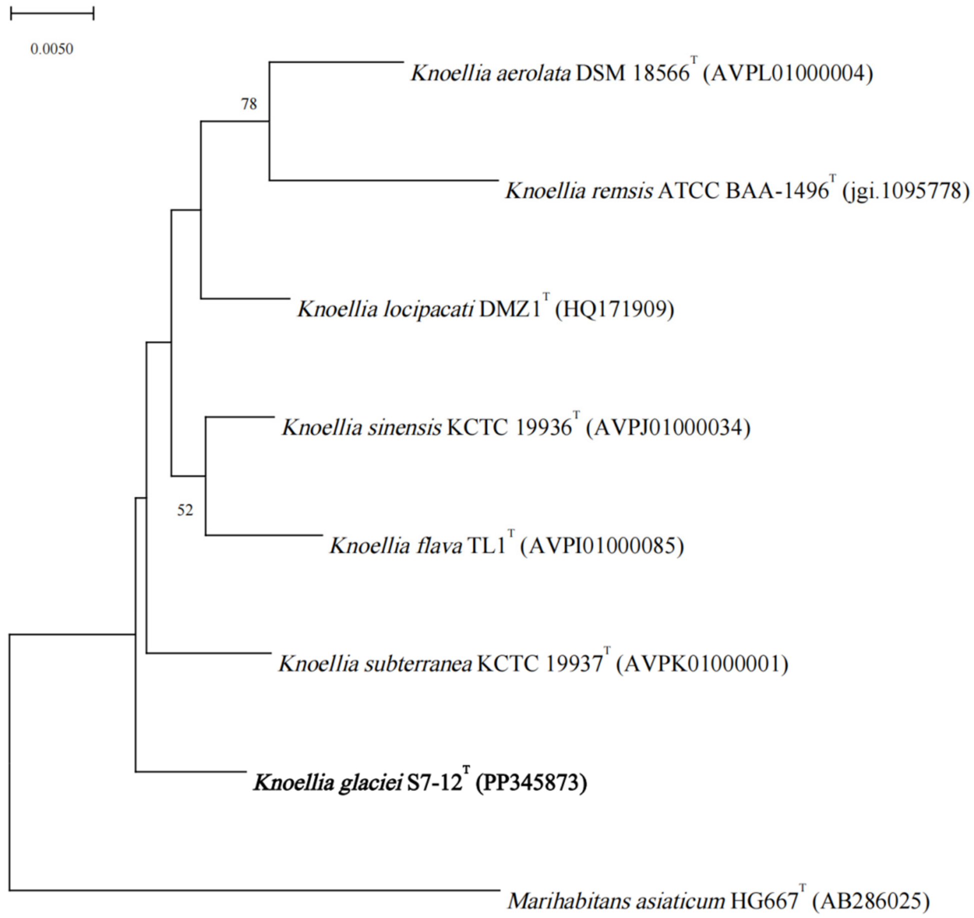

Figure S1. Minimum-evolution phylogenetic tree based on 16S rRNA gene sequences of the strain S7-12^{T}, and the type strains of other closely related species in the genus *Knoellia* and *Marihabitans*. *Marihabitans asiaticum* HG667^{T} (AB286025) was used as an outgroup. Bar, 0.005 substitutions per nucleotide position.

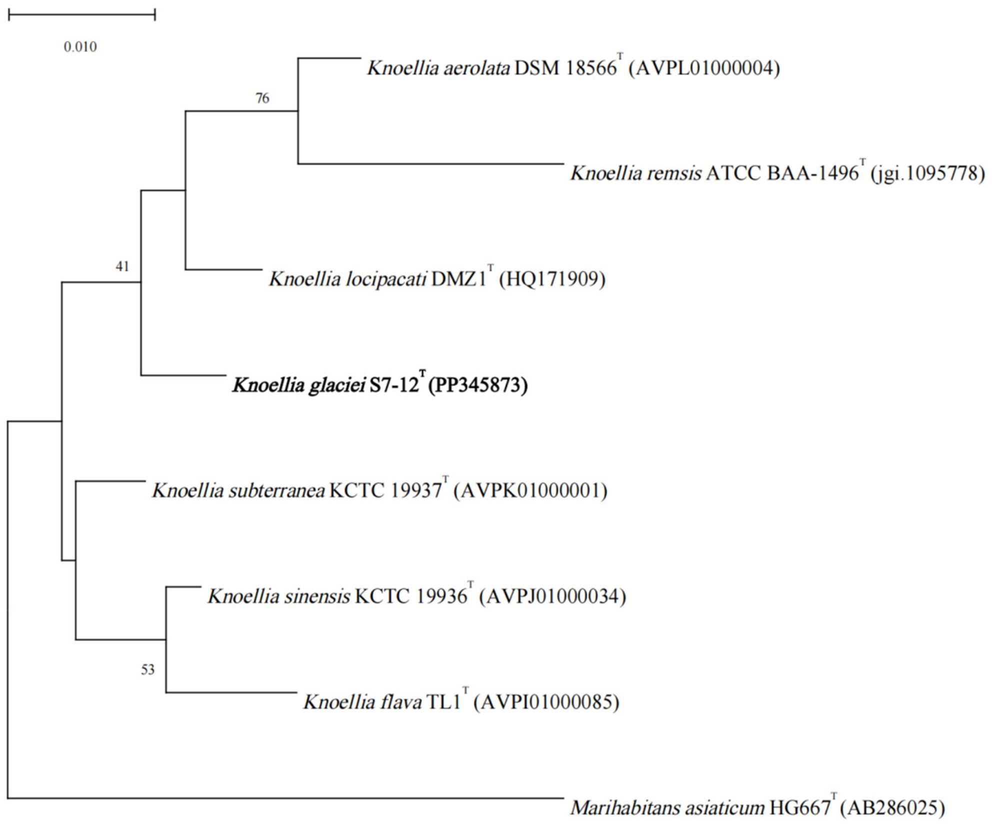

Figure S2. Maximum-likelihood phylogenetic tree based on 16S rRNA gene sequences of the strain S7-12^{T}, and the type strains of other closely related species in the genus *Knoellia* and *Marihabitans*. *Marihabitans asiaticum* HG667^{T} (AB286025) was used as an outgroup. Bar, 0.01 substitutions per nucleotide position.

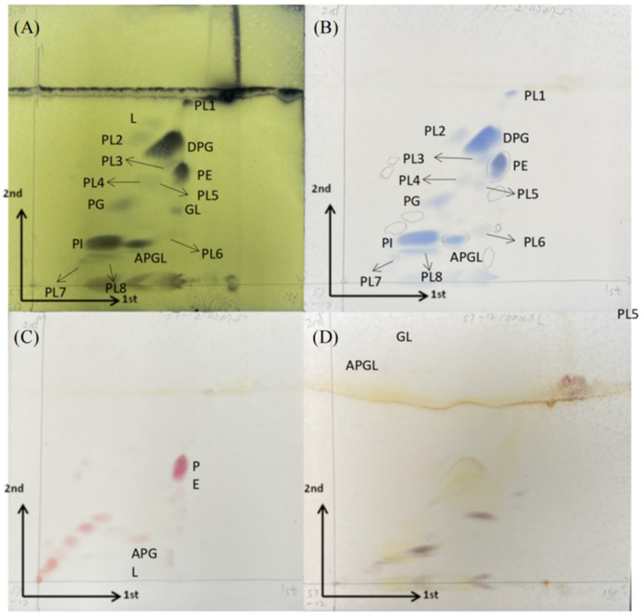

Figure S3. Polar lipids profile of strain S7-12^{T}. Total lipids were visualized after two-dimensional TLC and applying 5% ethanolic molybdatophosphoric acid. The solvent system was phosphomolybdic acid (**A**), molybdenum blue (**B**), indigohydrone (**C**), and α-naphthol (**D**) from left to right and top to bottom.

DPG, Diphosphatidylglycerol; PG, Phosphatidylglycerol; PE, Phosphatidylethanolamine; PI, Phosphatidylinositol; PL1-8, Phospholipids; GL, unidentified glycolipid; APGL, aminophosphoglycolipid.

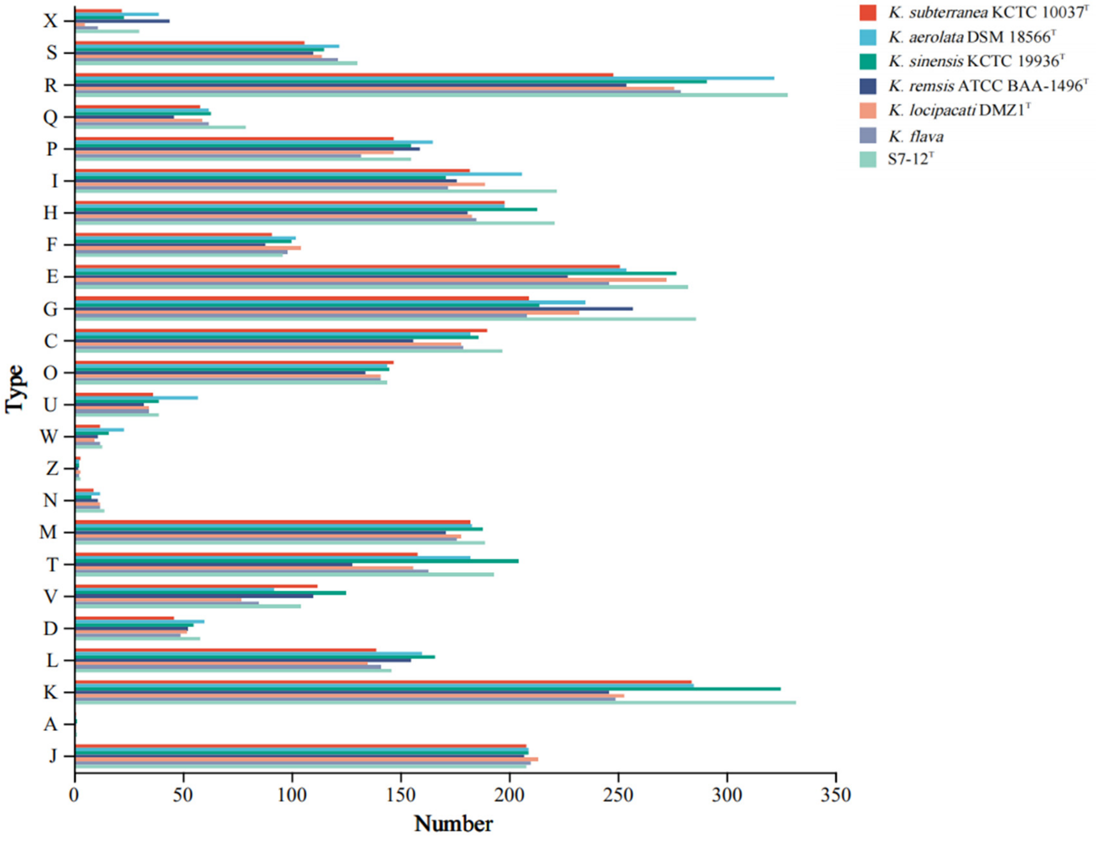

Figure S4. Comparison of COGs functional abundance between strain S7-12^{T} and similar strains in its genus.

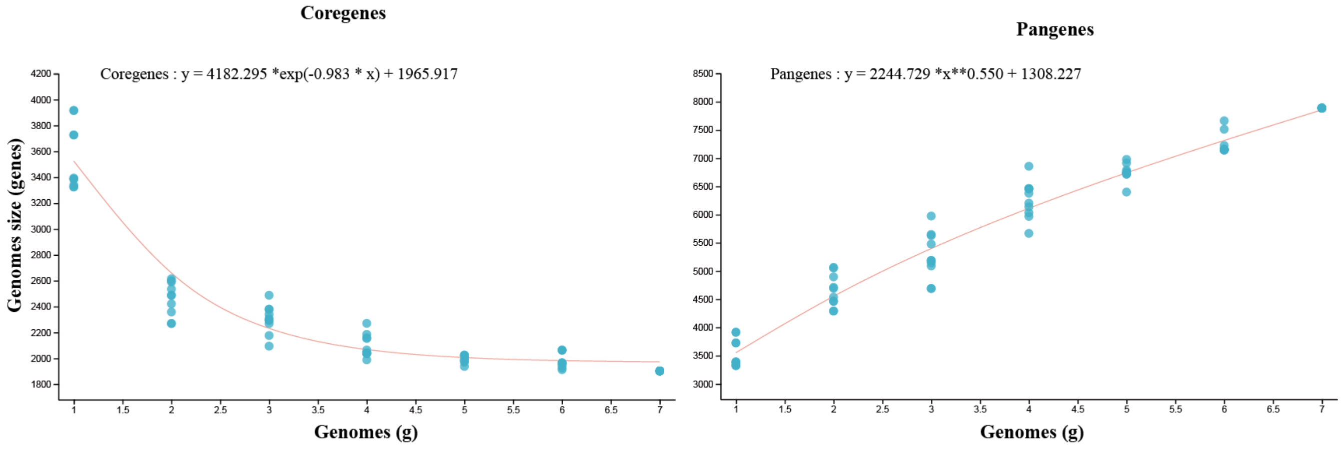

Figure S5. Characteristic curves of the pan-genome and core genome of S7-12^{T}.

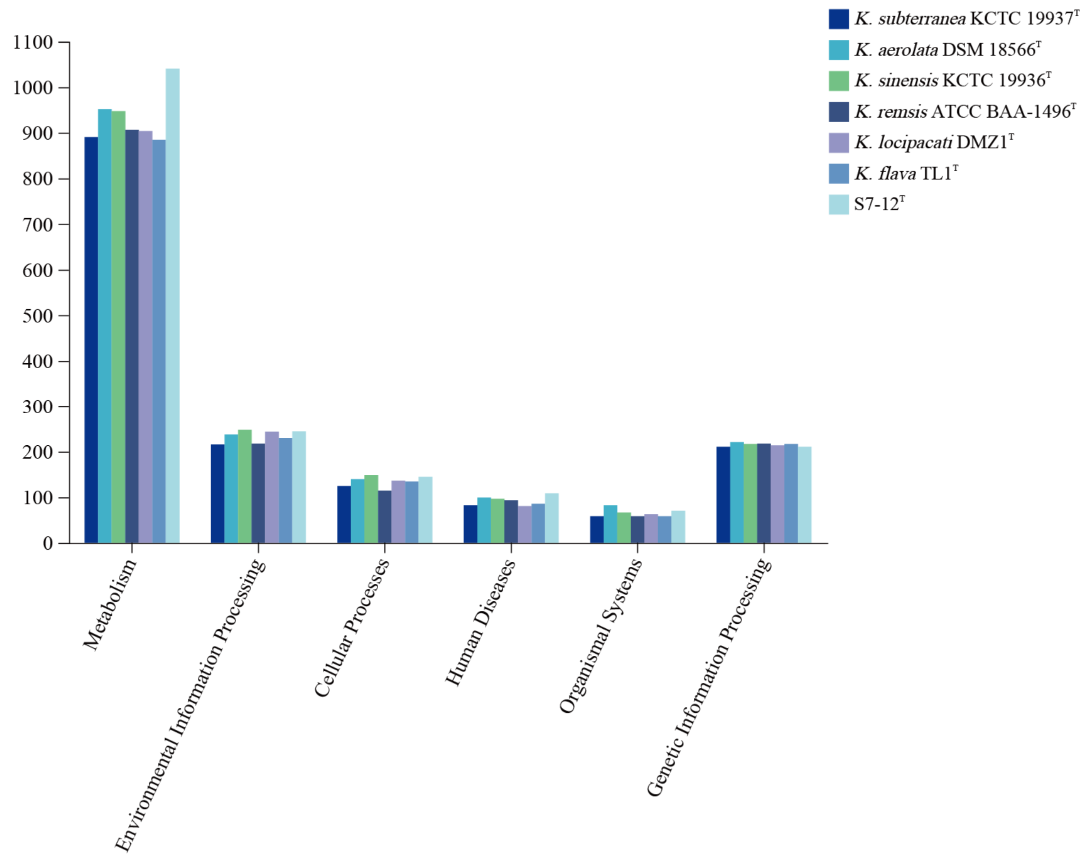

Figure S6. Phylogenetic functional analysis of all strains of the genus *Knoellia* using the KEGG database.

(A)

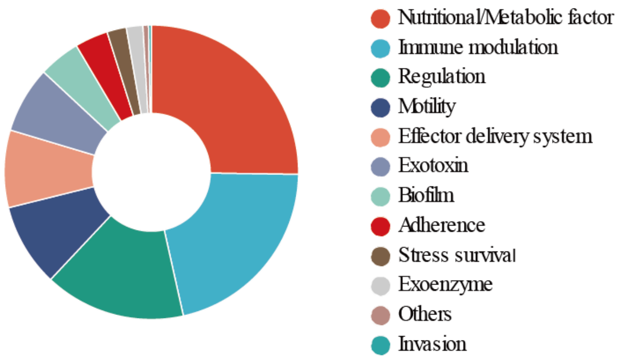

(B)

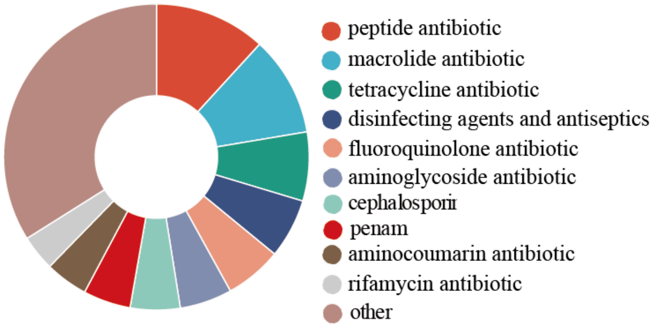

Figure S7. Virulence genes (A) and resistance gene prediction (B) of strain S7-12^{T}.

Tables

Table S1. General genomic characteristics comparison of strain S7-12^T, and its closely related species. Strains: 1. S7-12^T; 2. *K. flava* TL1^T; 3. *K. aerolata* DSM 18566^T; 4 . *K. locipacati* DMZ1^T; 5. *K. remsis* ATCC BAA-1496^T; 6. *K. sinensis* KCTC 19936^T; 7. *K. subterranea* KCTC 19937^T . NF, not found.

**Characteristics**	**1**	**2**	**3**	**4**	**5**	6	**7**
**Sequence status**	**Complete**	Draft	Draft	Draft	Draft	Draft	Draft
**Genome size (bp)**	**4163720**	3567168	4087970	3774873	3774843	4194304	3774873
**Contig N50 (kb)**	**3376213**	1123554	56.9	466.7	111.2	258.3	341.6
**CDS**	**3955**	3352	3813	3411	3385	3804	3418
**RNA**	**50**	50	52	55	49	48	47
**rRNA**	**6**	5	9	3	3	3	3
**tRNA**	**44**	45	43	52	46	45	44
**5S rRNA**	**2**	3	2	1	1	1	1
**16S rRNA**	**2**	1	5	1	1	1	1
**23S rRNA**	**2**	1	2	1	1	1	1
**GI**	**13**	4	4	4	5	7	4
**KEGG**	**1816**	1606	1728	1637	1605	1720	1579
**COG**	**3339**	2864	3175	2909	2866	3166	2923
**CARD**	**198**	163	233	184	173	232	176
**GO**	**2800**	2508	NF	NF	NF	2108	NF
**Swiss-Prot**	**2636**	2298	142	1039	1234	2317	1099
**CAZyme**	**109**	137	189	100	138	NF	99
**Secondary Metabolite**	**5**	3	7	3	NF	NF	NF
**DNA G+C content (mol%)**	**67.8**	71.5	64.8	65.1	65.4	65.6	69.0
**GenBank accession number**	**GCA_040 518285.1**	GCA_014 636255.1	GCA_000 768695.1	GCA_007 992835.1	GCA_003 002895.1	GCA_000 768705.1	GCA_000 768685.1

Table S2. The description of COG type.

COG Type	Type Description	Category
A	RNA processing and modification	INFORMATION STORAGE AND PROCESSING
C	Energy production and conversion	METABOLISM

D	Cell cycle control, cell division, chromosome partitioning	CELLULAR PROCESSES AND SIGNALING
E	Amino acid transport and metabolism	METABOLISM
F	Nucleotide transport and metabolism	METABOLISM
G	Carbohydrate transport and metabolism	METABOLISM
H	Coenzyme transport and metabolism	METABOLISM
I	Lipid transport and metabolism	METABOLISM
J	Translation, ribosomal structure and biogenesis	INFORMATION STORAGE AND PROCESSING
K	Transcription	INFORMATION STORAGE AND PROCESSING
L	Replication, recombination and repair	INFORMATION STORAGE AND PROCESSING
M	Cell wall/membrane/envelope biogenesis	CELLULAR PROCESSES AND SIGNALING
N	Cell motility	CELLULAR PROCESSES AND SIGNALING
O	Posttranslational modification, protein turnover, chaperones	CELLULAR PROCESSES AND SIGNALING
P	Inorganic ion transport and metabolism	METABOLISM
Q	Secondary metabolites biosynthesis, transport and catabolism	METABOLISM
R	General function prediction only	POORLY CHARACTERIZED
S	Function unknown	POORLY CHARACTERIZED
T	Signal transduction mechanisms	CELLULAR PROCESSES AND SIGNALING
U	Intracellular trafficking, secretion, and vesicular transport	CELLULAR PROCESSES AND SIGNALING
V	Defense mechanisms	CELLULAR PROCESSES AND SIGNALING
W	Extracellular structures	CELLULAR PROCESSES AND SIGNALING
X	Mobilome: prophages, transposons	CELLULAR PROCESSES AND SIGNALING
Z	Cytoskeleton	CELLULAR PROCESSES AND SIGNALING

Table S3. Features of the GIs found in the genome of S7-12^{T}

GI	Length (bp)	Total no. of gene	Hypothetical proteins	Predicted function
1	14275	21	13	Replication, recombination and repair; Transcription
2	42553	39	15	Amino acid transport and metabolism; Signal

				transduction mechanisms; Replication, recombination and repair; Cell wall/membrane/envelope biogenesis; Coenzyme transport and metabolism;General function prediction only; Secondary metabolites biosynthesis, transport and catabolism; Transcription; Defense mechanisms
3	16485	24	6	Cell wall/membrane/envelope biogenesis; Secondary metabolites biosynthesis, transport and catabolism;General function prediction only; Amino acid transport and metabolism; Coenzyme transport and metabolism; Replication, recombination and repair
4	14193	16	7	General function prediction only; Mobilome: prophages, transposons; Inorganic ion transport and metabolism
5	22641	21	6	Replication, recombination and repair; Transcription;Mobilome: prophages, transposons; Inorganic ion transport and metabolism; Cell cycle control, cell division, chromosome partitioning; Lipid transport and metabolism;Signal transduction mechanisms; Cell wall/membrane/envelope biogenesis
6	12208	9	2	Signal transduction mechanisms;Transcription; Cell cycle control, cell division, chromosome partitioning;Inorganic ion transport and metabolism;Cell wall/membrane/envelope biogenesis;Intracellular trafficking, secretion, and vesicular transport; Secondary metabolites biosynthesis, transport and catabolism; Energy production and conversion; Mobilome: prophages, transposons
7	14756	15	5	Defense mechanisms; Replication, recombination and repair
8	6254	8	4	Transcription; Mobilome: prophages, transposons; Coenzyme transport and metabolism; Cell cycle control, cell division, chromosome partitioning;Inorganic ion transport and metabolism;Cell wall/membrane/envelope biogenesis
9	8058	11	5	General function prediction only; Mobilome: prophages, transposons
10	9604	12	3	Energy production and conversion; Transcription; Replication, recombination and

				repair;Mobilome: prophages, transposons; Coenzyme transport and metabolism; Lipid transport and metabolism
11	14268	12	4	Intracellular trafficking, secretion, and vesicular transport; Mobilome: prophages, transposons; Signal transduction mechanisms; Energy production and conversion; Transcription;Signal transduction mechanisms
12	77767	71	25	Extracellular structures; Transcription; Defense mechanisms; Cell motility; Cell cycle control, cell division, chromosome partitioning; Intracellular trafficking, secretion, and vesicular transport; Inorganic ion transport and metabolism; Signal transduction mechanisms; Replication, recombination and repair; Cell wall/membrane/envelope biogenesis; Nucleotide transport and metabolism;General function prediction only; Translation, ribosomal structure and biogenesis; Secondary metabolites biosynthesis, transport and catabolism; Amino acid transport and metabolism; Lipid transport and metabolism; Carbohydrate transport and metabolism
13	7537	9	1	Secondary metabolites biosynthesis, transport and catabolism; Cell wall/membrane/envelope biogenesis;Cell cycle control, cell division, chromosome partitioning;Signal transduction mechanisms; General function prediction only; Transcription; Mobilome: prophages, transposons; Replication, recombination and repair
Total	260599	268	96	
